# Infectious Plant Diseases: Etiology, Current Status, Problems and Prospects in Plant Protection

**DOI:** 10.32607/actanaturae.11026

**Published:** 2020

**Authors:** P. A. Nazarov, D. N. Baleev, M. I. Ivanova, L. M. Sokolova, M. V. Karakozova

**Affiliations:** Belozersky Institute of Physico-Chemical Biology, Lomonosov Moscow State University, Moscow, 119991 Russia; Moscow Institute of Physics and Technology, Dolgoprudny, Moscow region, 141701 Russia; Federal Scientific Vegetable Center, VNIISSOK, Moscow region, 143080 Russia; All-Russian Scientific Research Institute of Medicinal and Aromatic Plants, Moscow, 117216 Russia; All-Russian Scientific Research Institute of Vegetable Growing, Branch of the Federal Scientific Vegetable Center, Vereya, Moscow region, 140153 Russia; Center of Life Sciences, Skolkovo Institute of Science and Technology, Moscow, 121205 Russia

**Keywords:** bacteria, fungi, viruses, pesticides, phytopathogen, selection, disease resistance, integrated pest management, biological control, agrotechnical control, plant immunity

## Abstract

In recent years, there has been an increase in the number of diseases caused by bacterial, fungal, and viral infections. Infections affect plants at different stages of agricultural production. Depending on weather conditions and the phytosanitary condition of crops, the prevalence of diseases can reach 70–80% of the total plant population, and the yield can decrease in some cases down to 80–98%. Plants have innate cellular immunity, but specific phytopathogens have an ability to evade that immunity. This article examined phytopathogens of viral, fungal, and bacterial nature and explored the concepts of modern plant protection, methods of chemical, biological, and agrotechnical control, as well as modern methods used for identifying phytopathogens.

## INTRODUCTION

A plant is considered to be susceptible to infection if environmental factors alter its physiological processes thus resulting in a disrupted structure, growth, functions, or other parameters. Plant diseases are classified as infectious and non-infectious depending on the nature of a causative agent. The symptoms of the disease may depend on its cause, nature, and the location of the impact site. The factors causing plant diseases can be of biotic and abiotic nature. Non-infectious diseases are caused by unfavorable growth conditions; they are not transmitted from a diseased plant to a healthy one. Infectious diseases, on the contrary, can spread from one susceptible host to another, since the infectious agent can reproduce in the plant or on its surface.

The signs of plant diseases include wilting, spotting (necrosis), mold, pustules, rot, hypertrophy and hyperplasia (overgrowth), deformation, mummification, discoloration, and destruction of the affected tissue. Wilting results from the loss of turgor pressure in the cells and tissues. It is caused by both abiotic and biotic factors. Spotting is mostly associated with the partial death of plant tissues due to biotic factors. Mold and pustules occur as a result of fungal damage to a plant. Rot leads to both the death of intracellular contents (bacterial wet or fungal dry rot) and destruction of the intercellular substance and cell membrane (fungal dry rot). Hypertrophy and hyperplasia represent an excessive growth and proliferation of the affected tissue caused by pathogens. Deformations (leaf wrinkling, twisting, and curling; threadlike leaves, fruit ugliness, and double-floweredness) can be caused by various biotic and abiotic factors due to an outflow of the products of photosynthesis, uneven intake of nutrients by the plant, or uneven growth of various tissue elements. In mummification, plant organs are damaged by the fungal mycelium, which leads to plant shrinkage, darkening, or compaction. Color changes usually occur due to chloroplast dysfunction and low content of chlorophyll in the leaves, which manifests itself in the light color of some leaf areas (mosaic discoloration) or the entire leaf (chlorosis) [1, 2].

Infectious agents can spread through the air, with water, be transmitted by animals, humans, and remain infectious for many months or years. The natural reservoirs of infectious agents are soil, water, and animals: especially insects.

**Fig. 1:**
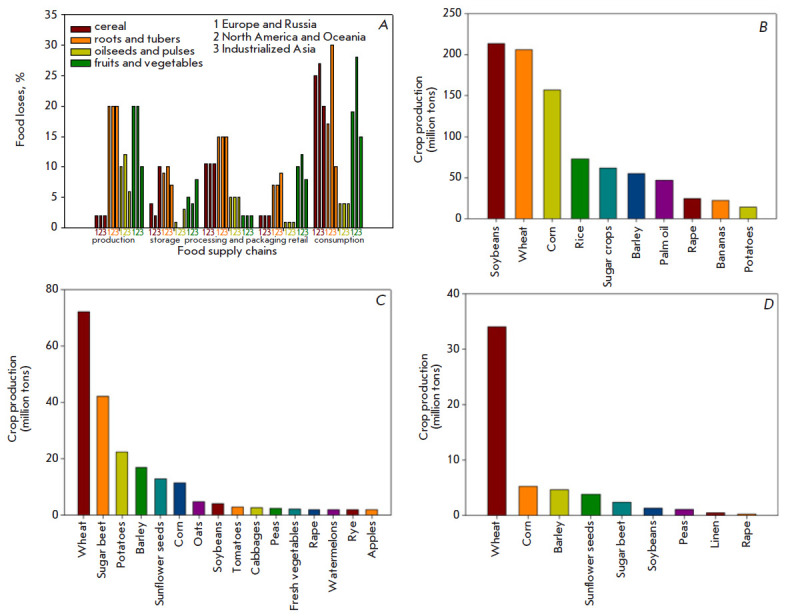
*A *- crop losses in industrialized countries (medium and high per capita income) at each stage of the production process, starting from cultivation and ending with consumption by households. The results present data for three regions: 1 – Europe (including Russia), 2 – North America and Oceania (USA, Canada, Australia, and New Zealand) and 3 – Industrial Asia (Japan, China, South Korea). Losses are calculated by weight as a percentage of the total mass of the product at the production stage [4]. *B *- top 10 most grown crops in the world (by import). *C *- the most grown plant crops in Russia. *D *- the main exported plant products from Russia [5]

Infectious plant diseases are mainly caused by pathogenic organisms such as fungi, bacteria, viruses, protozoa, as well as insects and parasitic plants [1]. With the development of agriculture, infectious plant diseases have become an increasingly significant factor affecting crop yield and economic efficiency. In the field environment, each plant cultivated as a monoculture has uniform conditions and requirements for planting, care, and harvesting, which leads to higher yields and lower production costs than in polyculture [3]. Over the past half century, the use of modern technologies, including cultivation of monocultures, has allowed us to reduce the amount of additional land needed for food production. However, growing the same crop in the same location year after year depletes the soil and renders it unable to ensure healthy plant growth. Another crucial issue is the susceptibility of monocultures to infectious diseases. Losses can amount to up to 30% even at the stage of storage, transportation, and distribution to the consumer (*Fig. 1*) [4, 5]. Therefore, it is necessary to arrest or prevent the development of infectious diseases at all stages of crop production: starting from seed handling technologies and ending with the delivery and storage of the product on store shelves and in consumers’ homes. This review summarizes existing data on the causes and pathogenetic mechanisms of infectious plant diseases caused by viruses, bacteria, and fungi that affect major agricultural crops, including cereals, vegetables, and industrial crops. The article considers the current status, as well as the problems and prospects of plant protection.

## PLANT IMMUNITY AND MECHANISMS FOR ITS EVASION

Plants typically are resistant to non-specific pathogens thanks to the presence of a waxy cuticle covering the epidermal cell layer and the constant synthesis of various antimicrobial compounds. Specific pathogens use a variety of strategies to penetrate plants, which often render such protection ineffective. Fungi can penetrate directly into epidermal cells or form hyphae over plant cells and between them, which does not require special structures or conditions. Meanwhile, bacterial and viral infections often require either damaged tissues, specialized structures (e.g., stomata) for entering the cell, or a specific carrier (vector). The latter is usually an insect, a fungus, or protozoa. How does plant infection with phytopathogens occur? In order to understand this, it is important to keep in mind that, unlike animals, plants rely on the innate immunity of each cell and systemic signals emanating from the sites of the infection and not on mobile defense cells and the somatic adaptive immune system. Moreover, an infection by pathogenic microorganisms is not always successful because of the structural changes in the cell wall or programmed cell death.

Plants have so-called trichomes: outgrowths of the epidermis that prevent pathogen growth and penetration. Trichomes may contain antimicrobial compounds or exert an inhibitory effect on the microbial hydrolytic enzymes involved in cell wall damage. The role of the cell wall cannot be overestimated: it is the first obstacle that pathogenic microorganisms must evade; successful protection at this line of defense is most effective against non-specific pathogens. The cell wall consists of cellulose microfibrils and hemicellulose; it is reinforced with lignin and contains a significant amount of proteins that perform structural and enzymatic functions [6]. The heterogeneity of the structure of the plant cell wall forces pathogens to use various strategies to penetrate it.

Antimicrobial plant compounds, which contain low-molecular-weight non-protein substances, are divided into two groups: phytoanticipins and phytoalexins. Phytoanticipins, such as saponins, phenylpropanoids, alkaloids, cyanogenic glycosides, and glucosinolates, are antimicrobial compounds pre-synthesized by plants. Phytoalexins are formed in response to a pathogenic attack and include various phenylpropanoids, alkaloids, and terpenes. An overlap between these groups of antimicrobial agents is explained by the fact that the phytoalexins of some plants can act as phytoanticipins in others [7]. In addition, small RNAs regulate the expression of a wide range of genes in plants and comprise natural immunity against viruses [8]. Plants can also absorb and process exogenous hairpin double-stranded RNAs (dsRNAs) to suppress the genes responsible for the life maintenance and virulence of viruses pathogenic to plants, fungi, and insects [9]. Aspartate-specific apoptotic proteases (phytaspases), which induce apoptosis, the process of programmed cell death, play an important role in plant defense [10].

Plants have two types of immune system. The first one uses transmembrane pattern recognition receptors that respond to slowly evolving microbial or pathogen-associated molecular patterns, while the second one acts mainly inside the cell using the polymorphic protein products encoded by most disease resistance (R) genes [11].

Plant R genes interact with the *avr *(avirulence) gene products of the corresponding pathogens. In the presence of the corresponding R gene encoding a receptor that triggers the defense response cascade, the receptor recognizes the *avr *gene product and the plant exhibits a resistance phenotype. For protection against bacterial, viral, and fungal infections, as well as against insects, plants encode only eight classes of the R gene products [12] that trigger the downstream reaction cascade, which indicates degeneracy of the plant immune system. The number of R genes in the genome can amount to about 100, which is clearly not enough to recognize all possible pathogens. Apparently, recognition of pathogens by the plant immune system is also of a degenerative nature [13].

The general mechanism of protection against pathogens is, apparently, as follows: during the first phase of an infection, receptors recognize pathogen-associated molecular structures (for instance, flagellin) and trigger an immune response to prevent colonization, which leads to the elimination of a non-specific infection. A specific pathogen produces effector molecules that interfere with the molecules of the immune response, which triggers the so-called effector-mediated susceptibility in susceptible plants. In resistant plants, the R gene products recognize effectors, with further formation of effector-mediated resistance, which can trigger a hypersensitivity (programmed cell death) response in the pathogen-infected area [13]. During the course of evolution, pathogens have developed several strategies to suppress plant defense responses, such as altering the programmed cell death pathway, inhibiting protective compounds in the cell wall, as well as changing the hormonal status of plants and the expression pattern of defense genes [14]. However, the products of R defense genes against a viral infection can trigger a series of responses at once. For instance, the defense against potato virus X first starts with the inhibition of viral replication in the absence of a hypersensitivity reaction, while overexpression of the *avr *gene induces a hypersensitivity reaction, which renders the plant extremely resistant to this virus [15].

Plants can develop the so-called acquired resistance if the infection that causes resistance in one part of the plant spreads to other parts. This fact indicates that the signaling molecules can move from the affected area to other cells and enhance immunity to the previously encountered pathogen. It should be noted that acquired resistance is not a *de novo *acquired resistance but an activation of the existing resistance genes in response to a pathogenic attack. The cells accumulate salicylic acid and the various proteins associated with pathogenesis (e.g., chitinase). Such acquired resistance is of a temporary nature and can be both systemic and local [16].

Symbiotic bacteria colonizing the rhizosphere antagonize soil pathogens through various mechanisms: siderophores suppress plant pathogens by competing for iron; antibiotics suppress competing microorganisms, while chitinases and glucanases lyse microbial cells. Moreover, as a result of symbiosis with bacteria, plants can develop another, extremely peculiar type of resistance: induced systemic resistance, which is also mediated by salicylic acid, ethylene, jasmonic acid, and lipopolysaccharides. In contrast to acquired systemic resistance, induced systemic resistance provides non-specific protection, has no dose-dependent correlation with the effect, does not affect the pathogen directly, and does not depend on the proteins associated with pathogenesis [16]. Instead, it is determined by the plant genotype and can cause changes in plant metabolism, leading to a general increase in resistance [16].

Thus, understanding the mechanisms of plant defense and the pathways utilized by phytopathogens to overcome that defense allows one to devise a systematic approach to plant protection.

## THE MOST SIGNIFICANT PHYTOPATHOGENS

**Viruses and viroids**

**Fig. 2:**
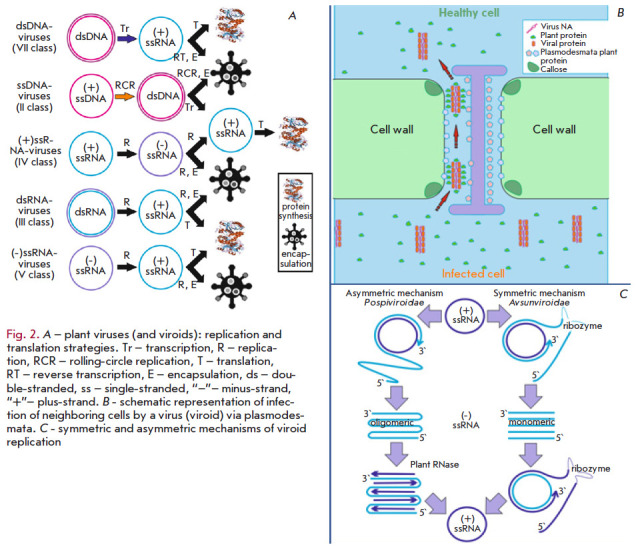
*A *– plant viruses (and viroids): replication and translation strategies. Tr – transcription, R – replication, RCR – rolling-circle replication, T – translation, RT – reverse transcription, E – encapsulation, ds – double-stranded, ss – single-stranded, “–”– minus-strand, “+”– plus-strand. *B *- schematic representation of infection of neighboring cells by a virus (viroid) via plasmodesmata. *C *- symmetric and asymmetric mechanisms of viroid replication

Viruses are non-cellular infectious agents that can only replicate in living cells. Viruses infect all types of organisms, from plants and animals to bacteria and archaea [17]. They can be integrated into the host’s genome and remain there as an inactive provirus or actively replicate and regulate the host’s biosynthesis processes. The suppression of viral gene transcription can lead to a latent infection [18]. Plant viruses mainly come in the form of single-stranded (ss) and double-stranded (ds) RNA viruses, as well as single-stranded and DNA-containing retroviruses [17]. Due to a wide diversity of their genetic material, the reproductive cycle and life pattern often vary from virus to virus (*Fig. 2A*). Viruses are composed of a nucleic acid molecule and a protective protein coat (capsid). Capsid can sometimes contain a combination of proteins and lipids, which form a lipoprotein membrane. The typical size of a plant virus is 30 nm [19].

The virion enters the cytoplasm of the plant cell via passive transport through wounds caused by mechanical damage to the cuticle and cell wall, since it is unable to pass through these structures on its own. Upon entering the cell, the virus uncoats. DNA-containing viruses also need to penetrate the nucleus in order to start transcription and mRNA synthesis. All viruses encode at least two types of proteins: replication proteins, which are required for the synthesis of nucleic acid, and structural proteins, which form the capsid. In some cases, there are also proteins that are responsible for virion motility; they ensure transport of virus particles between the plant cells. Viral replication proteins bind to cellular proteins to form a complex that produces multiple copies of the viral genome which interact with structural proteins to form new virions, which are then released from the cell. This is the standard viral life cycle.

Plant viruses can be transmitted vertically (from parents to offspring) and horizontally (from diseased plants to healthy ones). Viruses utilize small intercellular channels called plasmodesmata to penetrate neighboring cells (*Fig. 2B*). Viruses often express the proteins that ensure virion motility by modifying channels to facilitate the transmission of the infection to a neighboring cell [20]. This is how a local infection of a plant takes place. In order to infect an entire plant, a virus must enter its vascular system, where it then moves passively through the sieve tubes of the phloem with the flow of substances: this is how it can infect cells distant from the primary site of the infection [19, 20].

Some viruses are very stable and resistant to heat, can remain viable for a long time in plant cells and the products derived from them [21, 22], and can spread through passive mechanical transport from one plant to another [23]. However, most plant viruses actively spread from infected plants to healthy ones using a carrier organism (vector). Carriers are divided into a mechanical vector, in which the agent does not propagate, and a biological one, in which part of the viral life cycle takes place [24]. The main vectors of plant viruses are arthropods, nematodes, and fungi that feed on plants [25].

Plant viruses pose a serious threat to a wide range of crops, while the economic losses caused by viruses are second only to the losses caused by other pathogens [26]. Moreover, some viruses can infect more than 1,000 different plant species comprising more than 85 families [27]. In the majority of subtropical and tropical regions, a viral infection can lead to a loss of up to 98% of the crop [28]. Viruses manifest themselves in a different way depending on the stage of crop production: they can inflict colossal damage at the stage of crop growth, while at the stage of harvesting, storage, and transportation, the damage from a viral infection is minimal. It should be also noted that, in some cases, plants are found infected with viruses in the absence of any obvious symptoms [29].

The symptoms of viral diseases can be divided into five main types: growth suppression (reduced growth of the entire plant or its leading shoots); discoloration (mosaic, chlorotic rings, leaf chlorosis, variegation); deformations (leaf wrinkling, corrugation, threadlike leaves); necrosis; and impaired reproduction (flower sterility, parthenocarpy, shedding of flowers and ovaries) [2].

There is another type of infectious agents: viroids, which are circular RNAs that cause various diseases in plants and animals. Taxonomically, they belong to viruses (families *Pospiviroidae *and *Avsunviroidae*). In contrast to viruses, viroids lack a protein envelope (capsid) and present covalently linked ssRNA molecules 200–500 nucleotides long, which is 50-80 times shorter than the viral genome. Viroids do not encode proteins and cannot replicate autonomously. It is considered that the viroid can employ the DNA-dependent RNA polymerase, endoribonuclease, and DNA ligase 1 (which is usually silent) of the host cell for its replication [30]. Viroids replicate via a rolling-circle mechanism, with members of the families *Pospiviroidae *and *Avsunviroidae *replicating through an asymmetric and symmetric pathway, respectively (*Fig. 2C*). The molecular mechanism of the pathogenic action of viroids is not fully understood. It is believed that viroids can alter the phosphorylation state of gene products via binding to cellular kinases [31], affect the expression of the genes associated with growth, stress, development, and protection [32], induce the proteins associated with pathogenesis during an infection [33], cause post-transcriptional suppression of gene expression by RNA interference, impair splicing [34], and induce demethylation of rRNA genes. It is surprising that the substitution of one nucleotide at a certain position alters the pathogenicity of the viroid significantly [35]. The RNA molecule of *Pospiviroidae *family members has five domains: a central domain (C) containing the central, conserved region, which plays an important role in viroid replication; a pathogenicity domain (P) implicated in the manifestation of disease symptoms; a variable domain (V), which is, apparently, responsible for viroid adaptation; and the transport domains T1 and T2 (in cases of co-infection with two viroids, they can exchange with these domains, which can contribute to their evolution). Viroids of the family *Avsunviroidae *lack the central conserved region but contain the sequences involved in the formation of the ribozyme structures necessary for self-cleavage of RNA strands [36].

The main symptoms of viroid diseases are reduced growth of the entire plant or its parts, discoloration (chlorosis, anthocyanosis), and deformation of various organs [2].

Thus, viruses and viroids represent a rather large group of pathogens that cause plant diseases and can result in serious damage to crops in the absence of management and preventive measures, especially when infected at early stages of plant growth.

**Bacteria and phytoplasmas**

Bacteria are found almost everywhere and can be pathogenic to animals, plants, and fungi [37]. Bacterial genetic information is encoded in the DNA in the form of a chromosome; more than one chromosome can be found in a cell. A bacterial cell can contain extrachromosomal mobile genetic elements: plasmids that can carry important virulence factors or, on the contrary, biological control factors. Bacteria can also contain a prophage, which represents bacteriophage DNA integrated into the genome. Most bacteria divide by binary fission, usually with simultaneous duplication of both chromosomal DNA and extrachromosomal elements. Division of a bacterial cell requires the presence of the membrane potential [38]. Bacteria can contain more than one plasmid, since some of them can be lost during division. For instance, *Pantoea stewartii *can harbor up to 13 different plasmids [39]. Although bacteria usually transfer plasmids within their population [40], horizontal transfer of genetic information remains quite common in the prokaryotic world.

Bacteria have a cell membrane which separates the cytoplasm from the external environment. Bacteria are divided into Gram-positive and Gram-negative organisms depending on the cell wall structure [41]. The cell wall of Gram-positive bacteria consists of a membrane and a thick peptidoglycan layer. The main component of the latter is multilayered murein. Peptidoglycan also contains proteins, lipids, and teichoic and teichuronic acids. The cell wall of Gram-negative bacteria has two membranes with a peptidoglycan layer between them. The outer membrane contains lipopolysaccharides and porins but lacks teichoic and lipoteichoic acids.

**Fig. 3:**
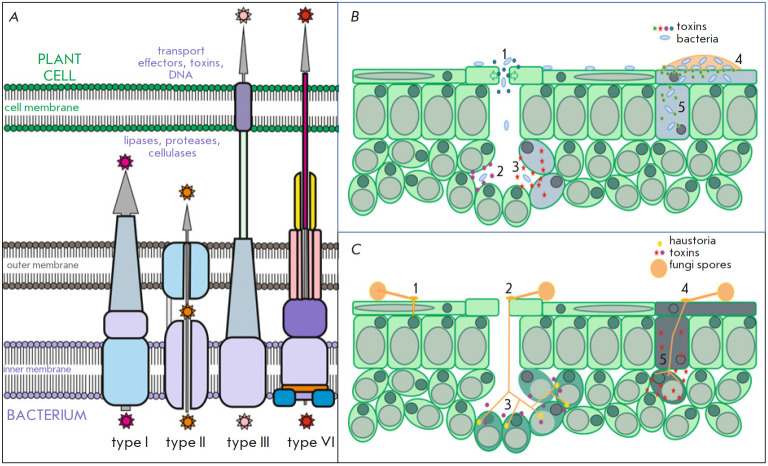
*A *– bacterial secretory systems that are used to infect plant cells and tissues. *B *- development of bacterial infection: 1 – penetration through the stomata due to phytotoxins, 2 – secretion of phytotoxins to modify the physiology, immune system, and metabolism of plants, 3 – secretion of phytotoxins for degradation of the cell wall and cytotoxic effect on plant cells, 4 – surface colonization and formation of biofilms, 5 – damage to plant cells due to ice nucleation and formation of crystals. *C *- development of fungal infection: 1 – penetration into an intact cell at the site of appressorium attachment through the combined effect of mechanical force and enzymes that destroy the plant cell wall, 2 – penetration of the fungus through stomata, 3 – secretion of phytotoxins to modify plant physiology, immune system, and metabolism in biotrophic fungi 4 – penetration of the fungus through the wound; 5 – secretion of phytotoxins for degradation of the cell wall and cytotoxic effect on plant cells

Due to the presence of a cell wall, bacteria need secretion systems to pump out xenobiotics, as well as release various proteins and virulence factors (*Fig. 3A*). The secretion systems are divided into several groups based on their structure. There are at least six different types of secretion systems typical of Gram-negative bacteria, four types found in Gram-positive bacteria, and two types present in both groups [42]. The secretion systems also play a key role in the virulence of phytopathogenic bacteria. It should be noted that, during the division of a bacterial cell, an asymmetry between mother and daughter cells can be observed, where the mother cell retains most of the secretion system transporters, while the daughter cell receives a smaller part of transporters and is forced to synthesize them *de novo *[43].

As a rule, phytopathogenic bacteria grow more slowly than non-pathogenic ones isolated from plants and have a temperature optimum of 20–30°C.

Bacterial pathogens contain several types of genes: virulence genes, which play a major role in infection and contribution to virulence, and disease-specific genes, which are important for disease manifestation (*Fig. 3B*). There are a series of genes that are required for host recognition, pathogen attachment to the plant surface, formation of infectious structures, as well as penetration and colonization of the host tissue. Pathogenic factors may either remain attached to the bacterial surface or can be released to the external environment. Pathogenic bacteria cause many serious plant diseases around the world, although not as many as fungi or viruses; however, the economic damage from bacterial diseases is relatively less severe than that from fungi and viruses [44]. Bacteria wreak havoc at all stages of crop production. Furthermore, due to the increase in the average annual temperature, there is reason to believe that the damage from bacterial spot and economic loses will only continue to grow in the coming years [45]. With an annual increase in the average daily temperature in summer of 3–4°C, the prevalence of bacterial diseases increases twofold, while the prevalence of plant infection grows by 30–50% [45].

There are two types of bacterial diseases: systemic bacterial blight (penetration of the pathogen in the plant’s vascular system, its further spread through the conductive bundles and adjacent tissues with disruption of the normal process of water consumption) and local bacterial blight (damage to the parenchymal tissues of individual plant organs). The main symptoms of bacterial diseases are wilting, necrosis, chlorosis, rot, overgrowth (galls), and scab.

Phytoplasmas and spiroplasmas are two groups of very small (about 1 μm in diameter) bacteria without a cell wall (they are separated from the external environment by a cytoplasmic membrane). They cause phytoplasmosis and growth retardation. Like mycoplasmas, a related genus of bacteria, phytoplasmas are apparently one of the most primitive and autonomously reproducing living organisms [46]. The genome of phytoplasmas is 0.5–1.3 million bp [47], while the genome of *Mycoplasma genitalium*, a model organism for studying the minimal genome, comprises 0.58 million bp [48]. Phytoplasmas exhibit gliding motility [49], while representatives of the genus *Spiroplasma *have a spiral shape and move in a twisting motion [50]. Cultivation of phytoplasmas in axenic cultures is quite difficult, which indicates their greater dependence on the host metabolism [51].

Phytoplasmosis significantly decreases both crop yield and its quality. Crop losses reach 40% for eggplants, 60% for tomatoes, 93% for pepper, 30–80% for potatoes, and 100% for cucumbers [52]. Plants with phytoplasmosis are characterized by such disorders of generative organs as virescence (greening of flowers and loss of normal pigmentation), phyllodia (transformation of part of a flower into a leaf-like formation), and proliferation (appearance of several “pseudo” flowers instead of one). In addition, phytoplasmosis can lead to the witches’ broom symptom (increased bushiness), dwarfism and wilting of plants, as well as leaf deformations. There is only one known case of positive phytoplasmosis, which leads to an economically useful effect: it is phytoplasmosis of poinsettia, a popular seasonal ornamental plant.

**Fungi**

Fungi are characteristic representatives of the domain Eukaryota. Unlike bacteria, they have a complex cell structure with a distinct nucleus and mitochondria. Fungal genome is much smaller than that of most eukaryotes but much larger than prokaryotic. Fungi have a cell wall, which usually consists of chitin, mannan, and chitosan, and also includes various proteins, lipids, and polyphosphates. Fungi form a mycelium: a system of thin branching hyphae, which sometimes lacks intercellular septa and forms a syncytium. Fungi are found in all ecological niches and can cause significant harm. Fungi appear to be evolutionarily much older than plants; the duration of their coexistence can be compared to the evolutionary age of higher plants [53]. About 80% of the plants present on our planet to date are symbiotic with fungi [54]. However, fungi sometimes disrupt the delicate balance of the mutually beneficial cooperation by turning into plant pathogens classified as biotrophs, hemibiotrophs, and necrotrophs. As a rule, pathogenic fungi enter plants through damaged leaves and stomata. However, in many cases, fungi secrete specific infectious structures and enzymes that destroy a plant’s cell wall (*Fig. 3C*). In the case of necrotrophs, which have a wide range of hosts, the host cells die quickly from the combined action of enzymes destroying the plant’s cell wall, reactive oxygen species, and/or toxins [55, 56]. Biotrophs, whose life cycle is associated with a living host cell, secrete effector molecules that suppress the plant’s immune system. These fungi exhibit specificity and interact with the host via special biotrophic hyphae in the interphase region where biomolecules synthesized by the plant are absorbed [57]. Fungi can develop specific outgrowths of hyphae, so-called apressoria, which provide attachment of the fungus to the substrate, thus allowing the pathogen to penetrate the cell wall using a combination of mechanical force and enzymes that degrade the plant’s cell wall. Haustoria move from the base of the appressorium through the destroyed areas and penetrate the lumen. As a rule, haustoria contain a large number of mitochondria and ribosomes with a well-developed endoplasmic reticulum; haurtorium is usually separated from the plant cell by invagination of the host plasmalemma [58]. At the same time, one can assume that an increased pressure of plant defense can cause a transition from biotrophy to necrotrophy [53].

Phytopathogenic fungi are the most dangerous plant pathogens to cause harm at all stages of crop production. The most common way to fight fungi is considered to be treatment with fungicides. The use of fungicides is associated with serious environmental and medical risks, namely the emergence of resistance and horizontal transfer of resistance genes, with the occurrence of species with multiple resistance [59]. At least 150 chemical compounds with different mechanisms of action are used as fungicides in world agriculture; however, there have been cases of resistance among various types of phytopathogens against almost all major classes of fungicides recorded to date [60].

The main symptoms of fungal diseases include wilting, spotting, mold (mycelium and sporulation of the fungus on the surface of affected organs), pustules (accumulation of fungal spores), overgrowth, deformations, mummification (shrinkage, darkening, and compaction of the infected tissue), and rot [2].

To date, more than 10,000 fungal species associated with plants have been discovered, and it is not surprising that fungal infections cause more harm than the diseases caused by other pathogenic microorganisms [61].

**Complex diseases**

Although it is believed that a plant disease is caused by one pathogen species or strain, microbes occur in nature mainly as part of complex multi-species consortia/communities. Most laboratory studies focus on individual strains grown in a pure culture. However, they cannot explain the complex course of certain plant diseases. Therefore, the diseases where more than one pathogen is involved are usually termed “complex” due to their complicated diagnosis and subsequent control [62]. Synergistic interactions can occur between viruses, bacteria, fungi, and different groups of pathogens. For instance, the synergism of virus–virus type is observed when cowpea is co-infected with cowpea mosaic virus and cucumber mosaic virus, with the severity of the disease and the degree of growth retardation being greater than in the case of infection with individual viruses [63]. Synergism of the type bacterium–bacterium, which exacerbates the disease severity, can be observed when tomato is co-infected with the bacteria *Pseudomonas corrugata *and *P. mediterranea*, which cause tomato pith necrosis [64]. Synergism of the type fungus–fungus occurs quite often; it causes complex diseases such as ascochyta blight complex of pea [65], mango malformation disease [66], etc. Brown apical necrosis of walnut resulting from the interaction of numerous pathogenic fungi and bacterium *Xanthomonas arboricola *represents an example of a synergistic interaction between different groups of pathogens [67]. Synergism between different pathogens resulting in more severe disease symptoms is more common than expected and may be crucial in understanding microbial pathogenesis and evolution, as well as further developing effective strategies of disease management [62].

**Fig. 4:**
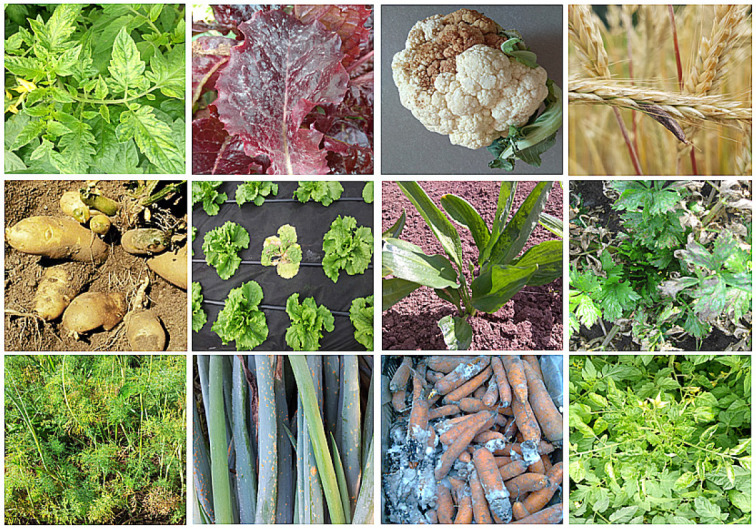
Infectious plant diseases. From left to right, top row: tomato mosaic virus, downy mildew of lettuce, bacterial blight of cauliflower, rye ergot, middle row: potato spindle tuber viroid (William M. Brown Jr, amended), lettuce bacterial blight, mixed viral infection on the ramson (cucumber mosaic virus, tobacco rattle virus, tobacco mosaic virus), Septoria blight of celery; bottom row: Fusarium blight of dill, onion rust, black rot (alternariosis) of carrots, and tomato leaf curl virus

Thus, phytopathogens are ubiquitous and cause various plant diseases (*Fig. 4*).

**Identification of phytopathogens**

Early diagnosis of plant diseases is a key factor that determines the timely use of protective measures and, as a result, determines the yield and quality of crop products. To date, in addition to conventional visual examination and the method of indicator plants, serological methods and methods based on DNA and RNA technologies are required in order to accurately identify plant diseases. The most common methods of serological diagnosis include enzyme immunoassay, immunoblotting, dot-blot hybridization, immunochromatography [68], and serologically specific electron microscopy [69]. Methods based on DNA detection include fluorescence *in situ *hybridization [70], various polymerase chain reaction (PCR) techniques, including nested PCR, cooperative PCR, multiplex PCR, real-time PCR, and DNA fingerprinting. There are also RNA-based approaches: isothermal amplification of nucleic acids [71], the AmpliDet RNA real-time diagnostic system [72], and reverse-transcription PCR. These methods allow for quick and accurate detection of the pathogen and identification of its taxonomic rank. Novel approaches for a more accurate and sensitive detection are now being developed. These are the next-generation sequencing and metagenomic analysis, two-hybrid analysis, phage display, as well as biosensor technologies based on electrochemistry and biophotonics [73]. Thus, modern methods allow for accurate identification of a phytopathogen even in the absence of infection symptoms.

**Integrated pest management (IPM)**

The system of managing the phytosanitary state of ecosystems using integrated methods of pest management to ensure the phytosanitary prosperity of the territory is effectively used in many countries [74].

IPM is based on the assessment of an acceptable level of pests for determining the pest threshold. A prerequisite for this is the constant monitoring of pests, quarantine measures and seed purity, as well as the selection of resistant varieties cultivated in the area. If the level of harmfulness is reached, then methods of mechanical and biological control are mostly applied; however, if necessary, chemical-control methods can be used in a responsible and targeted manner.

The costs of IPM and chemical management are practically comparable, while IPM provides longer duration of the effect, increases yields by 10–30%, improves product quality, reduces climate risks, and has a pronounced environmental upside [75].

**Seed reserves**

In the IPM paradigm, healthy planting material is a prerequisite for the effective use of the system. Unfortunately, the seeds of most plants often serve as reservoirs for various phytopathogens, and the infection can be located both on the surface of the seed and inside of it. There are several strategies for regulating the seed transmission of a pathogen existing to date: the use of pathogen-free seeds and the search for methods of pre-sowing seed treatment. The most effective way to combat fungi is considered to be treatment of seeds with fungicides. Contact fungicides are used to destroy pathogens on the seed surface, while translaminar fungicides can penetrate into the seed and destroy the pathogen inside of it. These agents must act delicately to avoid damaging the fetus [76]. In recent years, there have been various strategies developed to control the pathogens on seeds, including physical treatment (mechanical and thermal treatment, ultrasonic and ultraviolet light exposure), treatment with natural compounds and biological control agents, as well as substances inducing resistance [77].

About 11 million tons of agricultural seeds are sown in Russia annually. The volume rate of domestic seeds in the world’s cereal crops is 90%; it is 46% for corn, 43% for vegetables, 42% for soybeans, 32% for spring rape, and 26% for sunflower [78]. On the contrary, the volume rate of foreign seeds used in Russia varies from 30 to 90% depending on the culture, with the cost reaching 681,000 US dollars. The share of the seed business in the total sales of large agrochemical companies such as Syngenta, Bayer, DuPont, Dow, and Monsanto, is on the increase; they have acquired seed companies and comprehensively expanded their research on crop protection by developing and creating resistant varieties and hybrids using modern high-end and high-performance technologies, including genome editing [79].

**Plant breeding and bioengineering**

Modern plant breeding for resistance to pathogens utilizes approaches and methods of conventional and cell selection. The emergence of the complete genomic sequences of some economically important crops now makes it possible to effectively search for resistance genes, as well as the corresponding DNA markers. Today, genetic markers based on DNA polymorphism (RFLP, RAPD, AFLP, CAPS) and short tandem repeats (STRs, or SSRs), as well as DNA microarray technology Diversity Arrays Technology (DArT) [80, 81], are actively used. A long-term increase in plant resistance can be achieved by using gene pyramiding [82]; namely through the development of genetically engineered varieties and distant hybridization technology.

Modern biotechnology approaches are becoming increasingly important for the production of virus-resistant plant varieties and hybrids. Introduction of an antisense gene in the plant for its modification allows one to disrupt viral reproduction [83]. The gene encoding the protein that has an affinity for viral RNA and inhibits its replication is also inserted into the plant’s genome [84] to cause a delay in the expression of the transport protein or a modification of plasmodesma [85]. Constant expression of chitinase or lysozyme of bacteriophage T4 results in enhanced plant resistance to fungal and bacterial infections [86, 87]. Transgenic potato plants transcribing an RNA ribozyme that cleaves the RNA minus-strand of the spindle tuber viroid have been obtained [88].

New breeding methods to select varieties resistant to plant pathogens include powerful molecular tools for precise genetic modification, including the CRISPR/ Cas9 system, which allows for more accurate genome editing than the use of *Agrobacterium*-mediated transformation [89].

Agrotechnical control is a mandatory component of the IPM system. Adequate agricultural technology provides enhanced plant resistance to diseases and prevents massive infection by creating optimal conditions for plant growth and development. At the same time, crop rotation and selection of predecessors, the system of soil cultivation, fertilizers, dates of sowing and harvesting, as well as the destruction of weeds and post-harvest plant residues are of primary importance [90]. Placement of neighboring crops in the crop rotation and soil tillage are also essential [91]. Destroying post-harvest residues and weeds, which retain a large number of pathogens, while many weeds serve as reservoirs for them, is also of prime importance.

**Table:** The most significant phytopathogens

Viruses	Bacteria	Fungi
The world’s most significant phytopathogens		
Tobacco mosaic virus	Pseudomonas syringae	Magnaporthe oryzae
Tomato spotted wilt virus	Ralstonia solanacearum	Botrytis cinerea
Tomato yellow leaf curl virus	Agrobacterium tumefaciens	Puccinia spp.
Cucumber mosaic virus	Xanthomonas oryzae	Fusarium graminearum
Potato virus Y	Xanthomonas campestris	Fusarium oxysporum
Cauliflower mosaic virus	Xanthomonas axonopodis	Blumeria graminis
African cassava mosaic virus	Erwinia amylovora	Mycosphaerella graminicola
Plum pox virus	Xylella fastidiosa	Colletotrichum spp.
Brome mosaic virus	Dickeya dadantii	Ustilago maydis
Potato virus X	Dickeya solani	Melampsora lini
Citrus tristeza virus	Pectobacterium carotovorum	Phakopsora pachyrhizi
Barley yellow dwarf virus	Pectobacterium atrosepticum	Rhizoctonia solani
Potato leafroll virus	Clavibacter michiganensis	
Tomato bushy stunt virus		
The most significant phytopathogens in Russia		
Barley stripe mosaic virus	Candidatus Phytoplasma spp.	Alternaria solani
Wheat streak mosaic virus	Xanthomonas translucens	Fusarium avenaceum
Winter wheat Russian mosaic virus	Pseudomonas cichorii	Plasmopara halstedii
Oat Siberian mosaic virus	Rathayibacter tritici	Phytophthora infestans
Beet necrotic yellow vein virus	Pseudomonas fuscovaginae	Tilletia caries
Lettuce mosaic virus	Acidovorax citrulli	

**Chemical control**

Chemical control plays a crucial role in preventing losses associated with plant diseases, especially with the advent of numerous fungicides with selective toxicity, which expands possibilities for using them in targeted fashion.

The total cost of research, development, and registration of a new crop protection product rose from USD 152 million in 1995 to USD 286 million in 2014. Worldwide sales have been increasing by about 6.5% annually since 1999 [92]. There are more than 600 different chemical control agents on the market to date (fungicides, pesticides, herbicides, nematicides, molluscicides, rodenticides, and antibiotics), and the economic sector is now valued at more than USD 50 billion [93]. There are now strict regulations on the use of chemical pesticides; and many products have been taken off the market, banned or have failed to pass re-registration. For instance, six out of the ten major chemical control products used in 1968 are currently banned as household and agricultural pesticides in the United States.

**Biological control and alternative to antibiotics**

Modern agriculture is becoming an increasingly high-end and multidisciplinary industry with each passing year [94]. The uncontrolled use of herbicides leads to the appearance of populations of weeds that are resistant to them [95]. Although success in disease management mainly depends on crop resistance and the agricultural methods used, antibiotics such as gentamicin, oxolinic acid, oxytetracycline, and streptomycin are widely used in crop production [96]. The use of antibiotics in crop production is about 0.12%. However, in recent years, due to the widespread antibiotic resistance, more emphasis has been placed on alternative forms of combating phytopathogens. One such approach is the use of various methods of biological control [97]. Examples of biological control include the use of antagonist strains and antibiotic producers, bacteriophages, insects for weed control, and parasitic insects for controlling insect pests. For plant disease management, substances that are not themselves representatives of the groups of antibiotics or antimycotics, such as photosensitizers, bacteriophages, phagolysins, antimicrobial peptides, and antibiofilm agents [98], are used. They are especially useful if, in addition to antibacterial activity, they have other properties, e.g., the ability to reduce the level of reactive oxygen species or inhibit bacterial multidrug efflux pumps [99].

**The most significant plant pathogens**

Several years ago, *Molecular Plant Pathology *conducted a series of surveys among specialists in the field of molecular plant pathology, which allowed the journal to select the ten most significant phytopathogenic fungi [100], viruses [101], and bacteria [102] (*Table*).

One cannot but agree with such a choice. However, the structure of agricultural products and crops grown in Russia differs from global ones and is predominantly comprised of wheat, sugar beet, potatoes, barley, oats, sunflower, and corn and, thus, requires adjustments to the list of pathogens specific to these cultures [2, 65, 79, 103, 104]*. *

## CONCLUSION

With the advent of modern diagnostic approaches, genome editing and sequencing technologies, as well as microbiome and proteomic analysis methods, the study of the mechanisms and effect of phytopathogens on plants has moved to a multidisciplinary level. In this review, we have attempted to provide a comprehensive picture of the current state of pest management. However, to our deep regret, we could not consider many aspects of the interaction between plants and phytopathogens, such as damage by ice nucleation proteins, which cause the formation of ice crystals in plant cells [105] or the conserved nature of the sequences of effector molecules in bacteria: pathogens of humans, animals, and plants [106].

## Abbreviations

IPM: integrated pest management
RNA: ribonucleic acid,
DNA: deoxyribonucleic acid
